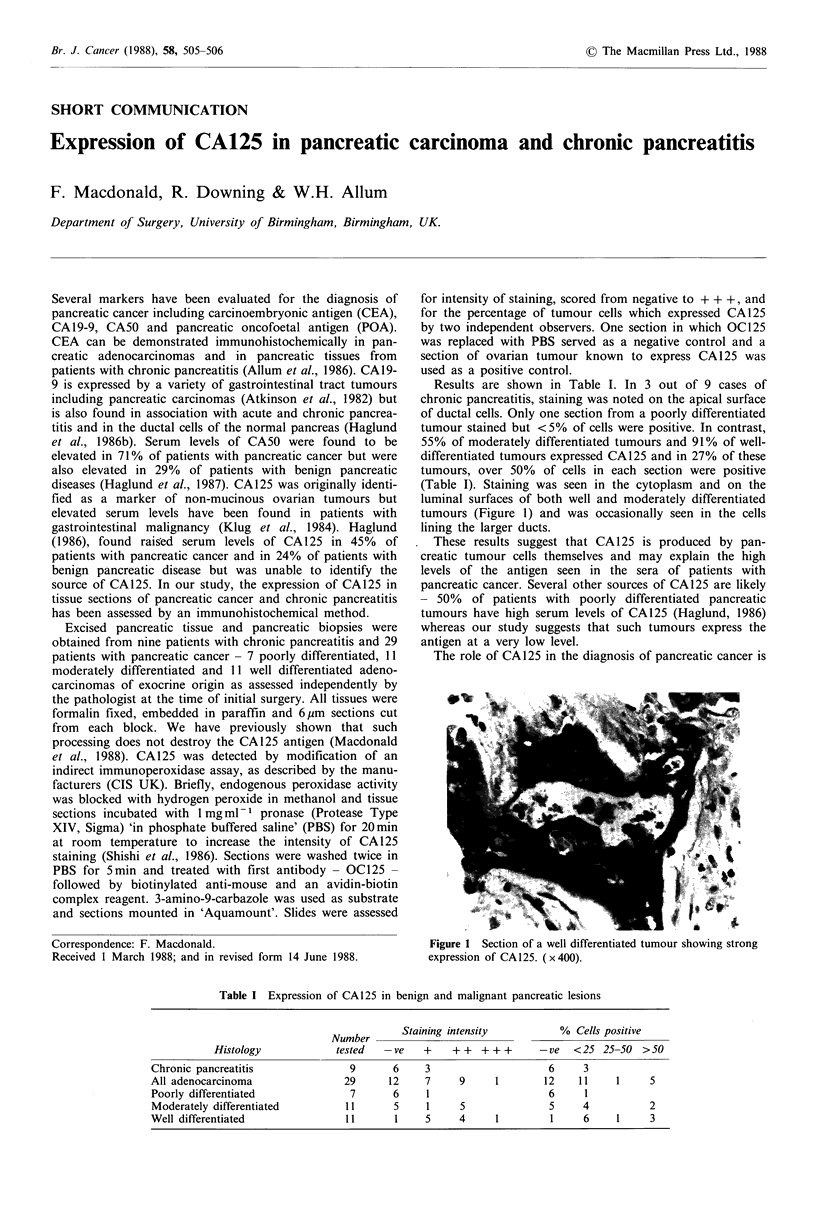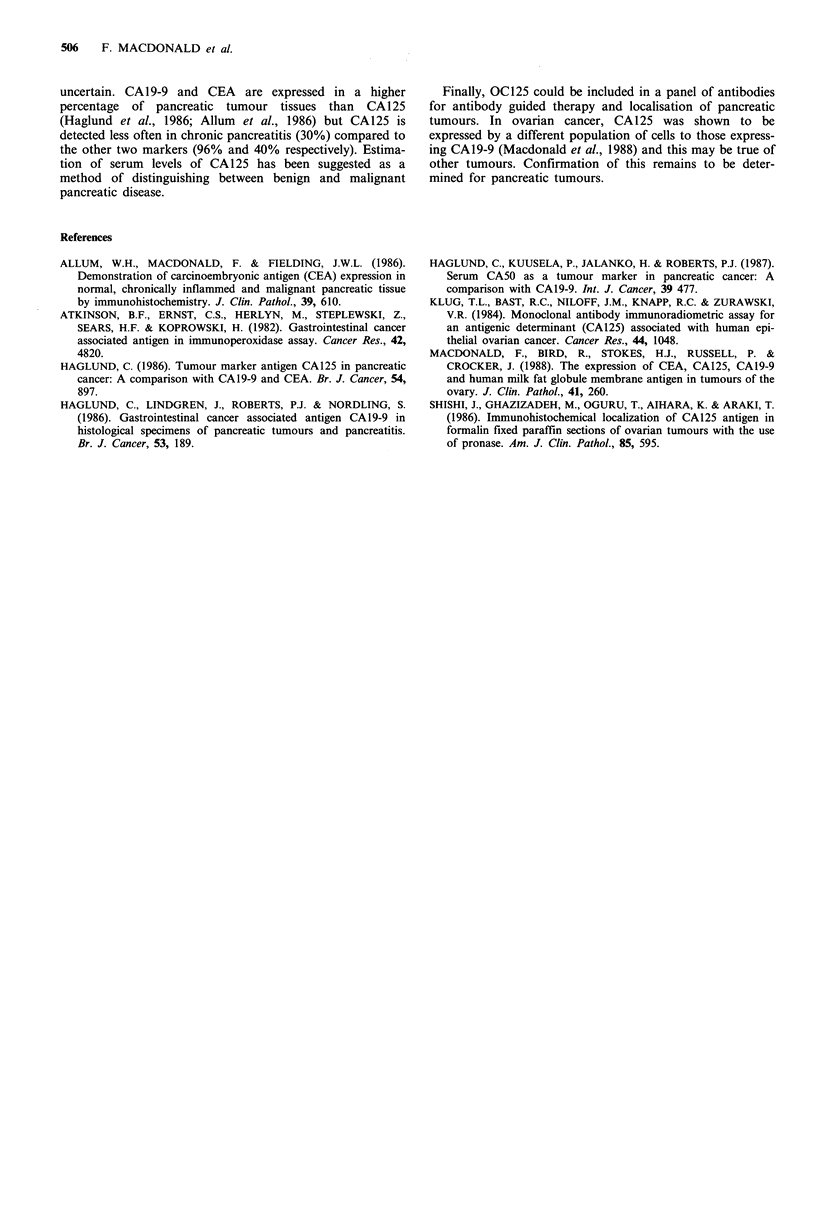# Expression of CA125 in pancreatic carcinoma and chronic pancreatitis.

**DOI:** 10.1038/bjc.1988.251

**Published:** 1988-10

**Authors:** F. Macdonald, R. Downing, W. H. Allum

**Affiliations:** Department of Surgery, University of Birmingham, UK.

## Abstract

**Images:**


Br. J. Cancer (1988), 58, 505-506 © The Macmillan Press Ltd., 1988

SHORT COMMUNICATION

### Expression of CA125 in pancreatic carcinoma and chronic pancreatitis

F. Macdonald, R. Downing & W.H. Allum

*Department of Surgery, University of Birmingham, Birmingham, UK.*

Several markers have been evaluated for the diagnosis of pancreatic cancer including carcinoembryonic antigen (CEA), CA19-9, CA50 and pancreatic oncofoetal antigen (POA). CEA can be demonstrated immunohistochemically in pancreatic adenocarcinomas and in pancreatic tissues from patients with chronic pancreatitis (Allum *et al.*, 1986). CA19-9 is expressed by a variety of gastrointestinal tract tumours including pancreatic carcinomas (Atkinson *et al.*, 1982) but is also found in association with acute and chronic pancreatitis and in the ductal cells of the normal pancreas (Haglund *et al.*, 1986b). Serum levels of CA50 were found to be elevated in 71% of patients with pancreatic cancer but were also elevated in 29% of patients with benign pancreatic diseases (Haglund *et al.*, 1987). CA125 was originally identified as a marker of non-mucinous ovarian tumours but elevated serum levels have been found in patients with gastrointestinal malignancy (Klug *et al.*, 1984). Haglund (1986), found raised serum levels of CA125 in 45% of patients with pancreatic cancer and in 24% of patients with benign pancreatic disease but was unable to identify the source of CA125. In our study, the expression of CA125 in tissue sections of pancreatic cancer and chronic pancreatitis has been assessed by an immunohistochemical method.

Excised pancreatic tissue and pancreatic biopsies were obtained from nine patients with chronic pancreatitis and 29 patients with pancreatic cancer – 7 poorly differentiated, 11 moderately differentiated and 11 well differentiated adenocarcinomas of exocrine origin as assessed independently by the pathologist at the time of initial surgery. All tissues were formalin fixed, embedded in paraffin and 6 $\mu$m sections cut from each block. We have previously shown that such processing does not destroy the CA125 antigen (Macdonald *et al.*, 1988). CA125 was detected by modification of an indirect immunoperoxidase assay, as described by the manufacturers (CIS UK). Briefly, endogenous peroxidase activity was blocked with hydrogen peroxide in methanol and tissue sections incubated with 1 mg ml$^{-1}$ pronase (Protease Type XIV, Sigma) 'in phosphate buffered saline' (PBS) for 20 min at room temperature to increase the intensity of CA125 staining (Shishi *et al.*, 1986). Sections were washed twice in PBS for 5 min and treated with first antibody – OC125 – followed by biotinylated anti-mouse and an avidin-biotin complex reagent. 3-amino-9-carbazole was used as substrate and sections mounted in 'Aquamount'. Slides were assessed for intensity of staining, scored from negative to + + +, and for the percentage of tumour cells which expressed CA125 by two independent observers. One section in which OC125 was replaced with PBS served as a negative control and a section of ovarian tumour known to express CA125 was used as a positive control.

Correspondence: F. Macdonald.
Received 1 March 1988; and in revised form 14 June 1988.

Results are shown in Table I. In 3 out of 9 cases of chronic pancreatitis, staining was noted on the apical surface of ductal cells. Only one section from a poorly differentiated tumour stained but <5% of cells were positive. In contrast, 55% of moderately differentiated tumours and 91% of well-differentiated tumours expressed CA125 and in 27% of these tumours, over 50% of cells in each section were positive (Table I). Staining was seen in the cytoplasm and on the luminal surfaces of both well and moderately differentiated tumours (Figure 1) and was occasionally seen in the cells lining the larger ducts.

These results suggest that CA125 is produced by pancreatic tumour cells themselves and may explain the high levels of the antigen seen in the sera of patients with pancreatic cancer. Several other sources of CA125 are likely – 50% of patients with poorly differentiated pancreatic tumours have high serum levels of CA125 (Haglund, 1986) whereas our study suggests that such tumours express the antigen at a very low level.

The role of CA125 in the diagnosis of pancreatic cancer is

**Figure 1** Section of a well differentiated tumour showing strong expression of CA125. (×400).

**Table I** Expression of CA125 in benign and malignant pancreatic lesions

| Histology | Number tested | Staining intensity –ve | + | ++ | +++ | % Cells positive –ve | <25 | 25–50 | >50 |
|---|---|---|---|---|---|---|---|---|---|
| Chronic pancreatitis | 9 | 6 | 3 | | | 6 | 3 | | |
| All adenocarcinoma | 29 | 12 | 7 | 9 | 1 | 12 | 11 | 1 | 5 |
| Poorly differentiated | 7 | 6 | 1 | | | 6 | 1 | | |
| Moderately differentiated | 11 | 5 | 1 | 5 | | 5 | 4 | | 2 |
| Well differentiated | 11 | 1 | 5 | 4 | 1 | 1 | 6 | 1 | 3 |

uncertain. CA19-9 and CEA are expressed in a higher percentage of pancreatic tumour tissues than CA125 (Haglund *et al.*, 1986; Allum *et al.*, 1986) but CA125 is detected less often in chronic pancreatitis (30%) compared to the other two markers (96% and 40% respectively). Estimation of serum levels of CA125 has been suggested as a method of distinguishing between benign and malignant pancreatic disease.

Finally, OC125 could be included in a panel of antibodies for antibody guided therapy and localisation of pancreatic tumours. In ovarian cancer, CA125 was shown to be expressed by a different population of cells to those expressing CA19-9 (Macdonald *et al.*, 1988) and this may be true of other tumours. Confirmation of this remains to be determined for pancreatic tumours.

**References**

ALLUM, W.H., MACDONALD, F. & FIELDING, J.W.L. (1986). Demonstration of carcinoembryonic antigen (CEA) expression in normal, chronically inflammed and malignant pancreatic tissue by immunohistochemistry. *J. Clin. Pathol.*, **39,** 610.

ATKINSON, B.F., ERNST, C.S., HERLYN, M., STEPLEWSKI, Z., SEARS, H.F. & KOPROWSKI, H. (1982). Gastrointestinal cancer associated antigen in immunoperoxidase assay. *Cancer Res.*, **42,** 4820.

HAGLUND, C. (1986). Tumour marker antigen CA125 in pancreatic cancer: A comparison with CA19-9 and CEA. *Br. J. Cancer*, **54,** 897.

HAGLUND, C., LINDGREN, J., ROBERTS, P.J. & NORDLING, S. (1986). Gastrointestinal cancer associated antigen CA19-9 in histological specimens of pancreatic tumours and pancreatitis. *Br. J. Cancer*, **53,** 189.

HAGLUND, C., KUUSELA, P., JALANKO, H. & ROBERTS, P.J. (1987). Serum CA50 as a tumour marker in pancreatic cancer: A comparison with CA19-9. *Int. J. Cancer*, **39** 477.

KLUG, T.L., BAST, R.C., NILOFF, J.M., KNAPP, R.C. & ZURAWSKI, V.R. (1984). Monoclonal antibody immunoradiometric assay for an antigenic determinant (CA125) associated with human epithelial ovarian cancer. *Cancer Res.*, **44,** 1048.

MACDONALD, F., BIRD, R., STOKES, H.J., RUSSELL, P. & CROCKER, J. (1988). The expression of CEA, CA125, CA19-9 and human milk fat globule membrane antigen in tumours of the ovary. *J. Clin. Pathol.*, **41,** 260.

SHISHI, J., GHAZIZADEH, M., OGURU, T., AIHARA, K. & ARAKI, T. (1986). Immunohistochemical localization of CA125 antigen in formalin fixed paraffin sections of ovarian tumours with the use of pronase. *Am. J. Clin. Pathol.*, **85,** 595.